# 2-Iodo-3-meth­oxy-6-methyl­pyridine

**DOI:** 10.1107/S1600536809050739

**Published:** 2009-11-28

**Authors:** Wenbo Guo, Xueqin Liu, Long Li, Dongsheng Deng

**Affiliations:** a College of Chemistry and Chemical Engineering, Luoyang Normal University, Luoyang 471022, People’s Republic of China; b Life Science Department, Luoyang Normal University, Luoyang 471022, People’s Republic of China; c Northwest Agriculture and Forest University, Yangling 712100, People’s Republic of China

## Abstract

The title compound, C_7_H_8_INO, which crystallizes with three independent mol­ecules in the asymmetric unit, was prepared by the reaction of 3-meth­oxy-6-methyl­pyridine with KI and I_2_ in tetra­hydro­furan solution. In the crystal structure, the three independent mol­ecules are arranged in a similar orientation with the three polar meth­oxy groups aligned on one side and the three non-polar methyl groups on the other side. The three mol­ecules, excluding methyl H atoms, are essentially planar, with r.m.s. deviations of 0.0141 (1), 0.0081 (1) and 0.0066 (2)Å. The three pyridine rings make dihedral angles of 58.09 (3) 66.64 (4) and 71.5 (3)°. The crystal structure features rather weak inter­molecular C—H⋯O hydrogen bonds, which link two mol­ecules into dimers, and short I⋯N contacts [4.046 (3) Å].

## Related literature

For C—C bond formation reactions, see: Vlad & Horvath (2002 ▶). For related structures, see: Bunker *et al.* (2009 ▶); Tahir *et al.* (2009 ▶).
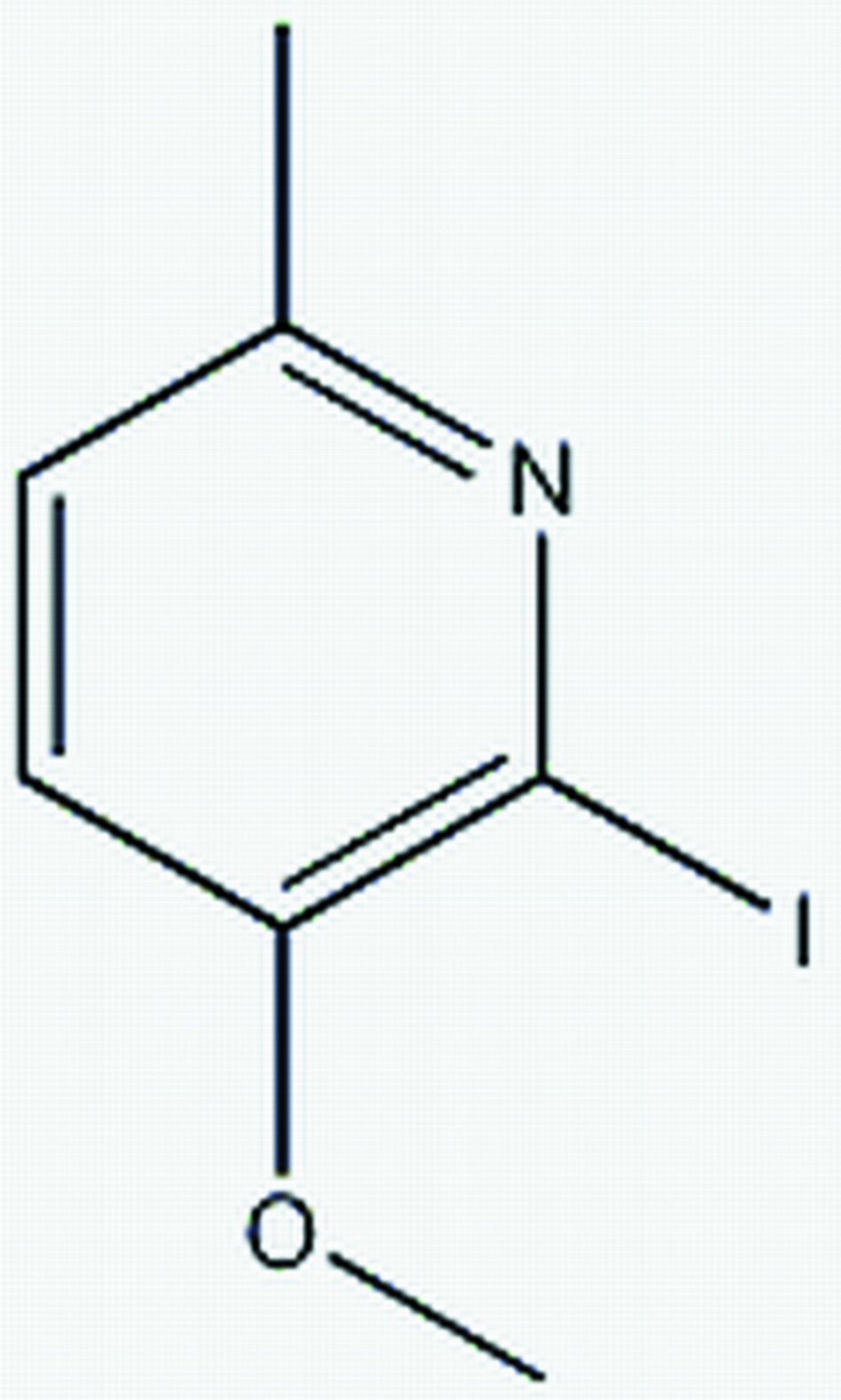


## Experimental

### 

#### Crystal data

C_7_H_8_INO
*M* *_r_* = 249.04
Triclinic, 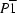

*a* = 7.7974 (9) Å
*b* = 10.8302 (12) Å
*c* = 16.2898 (18) Å
α = 106.093 (1)°
β = 90.633 (1)°
γ = 103.636 (1)°
*V* = 1280.2 (2) Å^3^
*Z* = 6
Mo *K*α radiation
μ = 3.69 mm^−1^
*T* = 296 K
0.20 × 0.14 × 0.13 mm

#### Data collection

Bruker APEXII CCD diffractometer
Absorption correction: multi-scan (*SADABS*; Sheldrick, 1996 ▶) *T*_min_ = 0.526, *T*_max_ = 0.646
9886 measured reflections
4737 independent reflections
3719 reflections with *I* > 2σ(*I*)
*R*_int_ = 0.026

#### Refinement

*R*[*F* ^2^ > 2σ(*F* ^2^)] = 0.029
*wR*(*F* ^2^) = 0.062
*S* = 1.01
4737 reflections
278 parameters
H-atom parameters constrained
Δρ_max_ = 0.56 e Å^−3^
Δρ_min_ = −0.68 e Å^−3^

### 

Data collection: *APEX2* (Bruker, 2004 ▶); cell refinement: *SAINT* (Bruker, 2004 ▶); data reduction: *SAINT*; program(s) used to solve structure: *SHELXS97* (Sheldrick, 2008 ▶); program(s) used to refine structure: *SHELXL97* (Sheldrick, 2008 ▶); molecular graphics: *SHELXTL* (Sheldrick, 2008 ▶) and *DIAMOND* (Brandenburg, 2006 ▶); software used to prepare material for publication: *SHELXTL* and *PLATON* (Spek, 2009 ▶).

## Supplementary Material

Crystal structure: contains datablocks global, I. DOI: 10.1107/S1600536809050739/bq2179sup1.cif

Structure factors: contains datablocks I. DOI: 10.1107/S1600536809050739/bq2179Isup2.hkl

Additional supplementary materials:  crystallographic information; 3D view; checkCIF report

## Figures and Tables

**Table 1:** Hydrogen-bond geometry (Å, °)

*D*—H⋯*A*	*D*—H	H⋯*A*	*D*⋯*A*	*D*—H⋯*A*
C14—H14*B*⋯O2^i^	0.96	2.56	3.429 (6)	151

Symmetry code: (i) 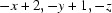.

**Fig. 1.**
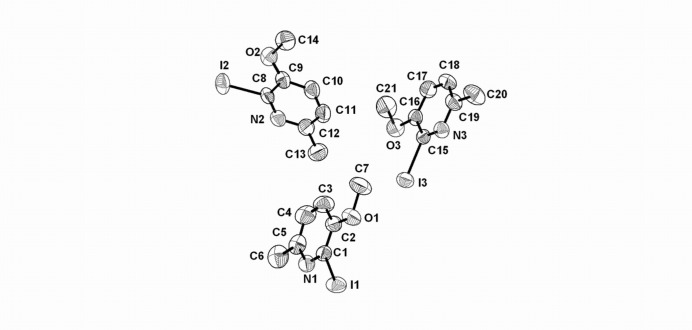
View of (I) (three molecule in the asymmetric unit) with atom numbering scheme and 30% probability displacement ellipsoids for non-hydrogen atoms.

**Fig. 2.**
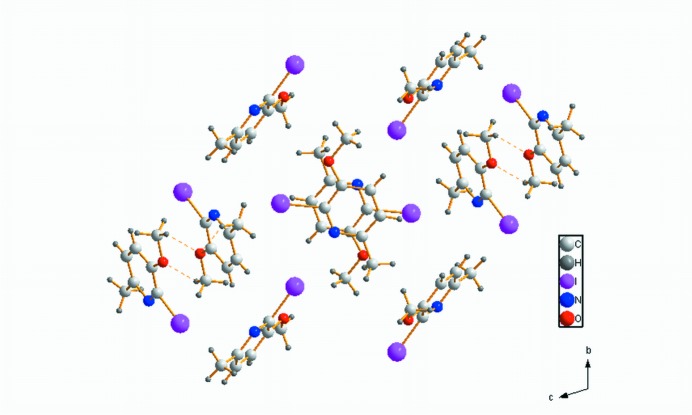
View of the dimers (C—H···O hydrogen bonds are indicated as broken lines).

## supplementary crystallographic information

### Comment

As is known, the Ullmann coupling reaction is an important C—C bond formation reaction. In this reaction, the halogen derivatives of aromatic compounds have been used as its reaction substrates (Vlad & Horvath. 2002). The reaction of 3-methoxy-6-methylpyridine with KI and I_2_ in the presence of NaHCO_3_ leads to iodo-substitution at position 2 of the pyridine ring with similar structure to the previous compound (Bunker *et al.* 2009), as shown by the X-ray study of the title compound (Fig. 1).

The asymmetric unit consists of three neutral C_7_H_8_INO molecules, in which the bond lengths and angles are within normal ranges (Bunker *et al.* 2009; Tahir *et al.* 2009). In the crystal structure, the three molecules are arranged in the similar orientation with the three polar methoxy groups aligning on one side and the three non-polar methyl groups siding on the other side. The pyridine ring 1 (C1-C5/N1) forms dihedral angles of 58.09 (3)° and 66.64 (4)°, respectively, with the pyridine ring 2 (C8-C12/N2) and the pyridine ring 3 (C15-19/N3). Rings 2 and 3 form a dihedral angle of 71.5 (3)°. Furthermore, the organic molecules, excluding methyl H atoms, are essentially planar, with r.m.s. deviations of 0.0141 (1), 0.0081 (1) and 0.0066 (2) Å. There are no strong halogen···halogen interactions in the structure, the shortest intermolecular I—I distances are 4.266 (2)Å. However, intermolecular C—H···O hydrogen bonds link the molecules into dimers, in which C14—H14B is donor and O2 is acceptor (Table 1, Fig. 2). This weak contacts may be effective in the stabilization of the structure.

### Experimental

To a solution of 3-methoxy-6-methylpyridine (4.00 g, 30 mmol) in THF 30 ml was added 10 ml water containing 2.69 g NaHCO_3_ (32 mmol). The mixture was stirred for 30 minutes. Under ice bath, the resulting solution was added dropwise a solution of I_2_ (8.12 g, 32 mmol) and KI (5.31 g, 32 mmol) in water (75 ml). The mixture was then stirred 72 h at room temperature, and treated with 15° solution of sodium thiosulfate, then filtered. The resulting white solid was rinsed with ice water, and dried under vacuum to afford 2-iodo-3-methoxy-6-methylpyridine, 6.48 g, 89.7° yield. The crystalline compound was obtained through the slow volatilization of ethyl acetate containing the title compound.

### Refinement

All H atoms were positioned geometrically and treated as riding, with C—H bond lengths constrained to 0.93 Å (aromatic CH), 0.96 Å (methyl CH_3_), and with *U*ĩso~(H) = 1.2U_eq_(C) or 1.5U_eq_(methyl).

### Crystal data

**Table:**

C_7_H_8_INO	*Z* = 6
*M**_r_* = 249.04	*F*(000) = 708
Triclinic, *P*1	*D*_x_ = 1.938 Mg m^−^^3^
Hall symbol: -P 1	Mo *K*α radiation, λ = 0.71073 Å
*a* = 7.7974 (9) Å	Cell parameters from 3296 reflections
*b* = 10.8302 (12) Å	θ = 2.7–23.1°
*c* = 16.2898 (18) Å	µ = 3.69 mm^−^^1^
α = 106.093 (1)°	*T* = 296 K
β = 90.633 (1)°	Block, colorless
γ = 103.636 (1)°	0.20 × 0.14 × 0.13 mm
*V* = 1280.2 (2) Å^3^	

### Data collection

**Table:**

Bruker APEXII CCD diffractometer	4737 independent reflections
Radiation source: fine-focus sealed tube	3719 reflections with *I* > 2σ(*I*)
graphite	*R*_int_ = 0.026
phi and ω scans	θ_max_ = 25.5°, θ_min_ = 2.6°
Absorption correction: multi-scan (*SADABS*; Sheldrick, 1996)	*h* = −9→9
*T*_min_ = 0.526, *T*_max_ = 0.646	*k* = −12→13
9886 measured reflections	*l* = −19→19

### Refinement

**Table:**

Refinement on *F*^2^	Secondary atom site location: difference Fourier map
Least-squares matrix: full	Hydrogen site location: inferred from neighbouring sites
*R*[*F*^2^ > 2σ(*F*^2^)] = 0.029	H-atom parameters constrained
*wR*(*F*^2^) = 0.062	*w* = 1/[σ^2^(*F*_o_^2^) + (0.0183*P*)^2^ + 0.8733*P*] where *P* = (*F*_o_^2^ + 2*F*_c_^2^)/3
*S* = 1.01	(Δ/σ)_max_ = 0.001
4737 reflections	Δρ_max_ = 0.56 e Å^−^^3^
278 parameters	Δρ_min_ = −0.68 e Å^−^^3^
0 restraints	Extinction correction: *SHELXS97* (Sheldrick, 2008), Fc^*^=kFc[1+0.001xFc^2^λ^3^/sin(2θ)]^-1/4^
Primary atom site location: structure-invariant direct methods	Extinction coefficient: 0.0067 (3)

### Special details

**Table:**

Geometry. All e.s.d.'s (except the e.s.d. in the dihedral angle between two l.s. planes) are estimated using the full covariance matrix. The cell e.s.d.'s are taken into account individually in the estimation of e.s.d.'s in distances, angles and torsion angles; correlations between e.s.d.'s in cell parameters are only used when they are defined by crystal symmetry. An approximate (isotropic) treatment of cell e.s.d.'s is used for estimating e.s.d.'s involving l.s. planes.
Refinement. Refinement of *F*^2^ against ALL reflections. The weighted *R*-factor *wR* and goodness of fit *S* are based on *F*^2^, conventional *R*-factors *R* are based on *F*, with *F* set to zero for negative *F*^2^. The threshold expression of *F*^2^ > σ(*F*^2^) is used only for calculating *R*-factors(gt) *etc*. and is not relevant to the choice of reflections for refinement. *R*-factors based on *F*^2^ are statistically about twice as large as those based on *F*, and *R*- factors based on ALL data will be even larger.

### Fractional atomic coordinates and isotropic or equivalent isotropic displacement parameters (Å^2^)

**Table:**

	*x*	*y*	*z*	*U*_iso_*/*U*_eq_	
C1	0.2655 (6)	0.5314 (4)	0.5640 (3)	0.0522 (10)	
C2	0.3438 (6)	0.6096 (5)	0.5130 (3)	0.0581 (11)	
C3	0.3160 (7)	0.5495 (6)	0.4251 (3)	0.0776 (15)	
H3	0.3633	0.5957	0.3870	0.093*	
C4	0.2179 (7)	0.4212 (6)	0.3958 (3)	0.0801 (15)	
H4	0.2010	0.3797	0.3371	0.096*	
C5	0.1440 (6)	0.3525 (5)	0.4509 (3)	0.0652 (13)	
C6	0.0322 (8)	0.2131 (5)	0.4196 (3)	0.0905 (17)	
H6A	0.0896	0.1550	0.4380	0.136*	
H6B	0.0167	0.1866	0.3581	0.136*	
H6C	−0.0813	0.2084	0.4425	0.136*	
C7	0.5208 (8)	0.8131 (5)	0.4975 (4)	0.0900 (17)	
H7A	0.5924	0.7653	0.4600	0.135*	
H7B	0.5938	0.8961	0.5321	0.135*	
H7C	0.4311	0.8294	0.4639	0.135*	
C8	0.5213 (6)	0.3742 (4)	0.0739 (2)	0.0479 (10)	
C9	0.6383 (6)	0.4983 (4)	0.0899 (3)	0.0500 (10)	
C10	0.5793 (7)	0.6050 (4)	0.1397 (3)	0.0627 (12)	
H10	0.6521	0.6906	0.1541	0.075*	
C11	0.4127 (7)	0.5816 (5)	0.1669 (3)	0.0671 (13)	
H11	0.3725	0.6521	0.2004	0.081*	
C12	0.3034 (6)	0.4552 (5)	0.1456 (3)	0.0596 (11)	
C13	0.1181 (7)	0.4251 (5)	0.1713 (3)	0.0791 (15)	
H13A	0.0364	0.4120	0.1233	0.119*	
H13B	0.1029	0.4979	0.2176	0.119*	
H13C	0.0959	0.3460	0.1894	0.119*	
C14	0.9152 (7)	0.6345 (4)	0.0738 (3)	0.0765 (15)	
H14A	0.8591	0.6908	0.0527	0.115*	
H14B	1.0216	0.6276	0.0458	0.115*	
H14C	0.9438	0.6715	0.1345	0.115*	
C15	0.1107 (5)	0.9067 (4)	0.2665 (2)	0.0450 (9)	
C16	0.2909 (5)	0.9519 (4)	0.2630 (3)	0.0483 (10)	
C17	0.3429 (6)	1.0324 (4)	0.2107 (3)	0.0624 (12)	
H17	0.4627	1.0660	0.2063	0.075*	
C18	0.2179 (7)	1.0628 (4)	0.1653 (3)	0.0646 (12)	
H18	0.2528	1.1183	0.1308	0.077*	
C19	0.0416 (6)	1.0116 (4)	0.1705 (3)	0.0594 (11)	
C20	−0.1023 (7)	1.0386 (6)	0.1207 (4)	0.0880 (17)	
H20A	−0.1829	0.9561	0.0903	0.132*	
H20B	−0.0505	1.0840	0.0807	0.132*	
H20C	−0.1652	1.0928	0.1596	0.132*	
C21	0.5866 (6)	0.9547 (5)	0.3028 (4)	0.0871 (17)	
H21A	0.6098	0.9276	0.2436	0.131*	
H21B	0.6499	0.9151	0.3351	0.131*	
H21C	0.6248	1.0495	0.3247	0.131*	
I1	0.30556 (5)	0.61072 (3)	0.698542 (19)	0.07157 (13)	
I2	0.60344 (4)	0.20520 (3)	0.00399 (2)	0.06819 (12)	
I3	0.01400 (4)	0.78330 (3)	0.344715 (19)	0.05933 (11)	
N1	0.1683 (5)	0.4079 (4)	0.5349 (2)	0.0568 (9)	
N2	0.3602 (5)	0.3508 (3)	0.0991 (2)	0.0538 (9)	
N3	−0.0123 (4)	0.9329 (3)	0.2220 (2)	0.0526 (9)	
O1	0.4377 (4)	0.7353 (3)	0.5523 (2)	0.0727 (9)	
O2	0.7978 (4)	0.5059 (3)	0.05666 (19)	0.0619 (8)	
O3	0.3999 (4)	0.9124 (3)	0.3106 (2)	0.0648 (8)	

### Atomic displacement parameters (Å^2^)

**Table:**

	*U*^11^	*U*^22^	*U*^33^	*U*^12^	*U*^13^	*U*^23^
C1	0.059 (3)	0.060 (3)	0.043 (2)	0.024 (2)	0.006 (2)	0.016 (2)
C2	0.058 (3)	0.071 (3)	0.058 (3)	0.027 (2)	0.010 (2)	0.028 (2)
C3	0.080 (4)	0.103 (4)	0.060 (3)	0.028 (3)	0.011 (3)	0.036 (3)
C4	0.096 (4)	0.098 (4)	0.043 (3)	0.029 (4)	0.001 (3)	0.012 (3)
C5	0.065 (3)	0.071 (3)	0.056 (3)	0.024 (3)	−0.006 (2)	0.005 (2)
C6	0.106 (4)	0.082 (4)	0.069 (3)	0.022 (3)	−0.012 (3)	0.000 (3)
C7	0.091 (4)	0.091 (4)	0.104 (4)	0.014 (3)	0.018 (3)	0.061 (4)
C8	0.062 (3)	0.040 (2)	0.045 (2)	0.017 (2)	−0.005 (2)	0.0143 (18)
C9	0.061 (3)	0.042 (2)	0.046 (2)	0.013 (2)	−0.008 (2)	0.0104 (19)
C10	0.081 (3)	0.043 (2)	0.060 (3)	0.016 (2)	0.002 (3)	0.008 (2)
C11	0.092 (4)	0.058 (3)	0.058 (3)	0.034 (3)	0.009 (3)	0.014 (2)
C12	0.065 (3)	0.069 (3)	0.056 (3)	0.028 (3)	0.005 (2)	0.026 (2)
C13	0.076 (4)	0.100 (4)	0.074 (3)	0.035 (3)	0.017 (3)	0.035 (3)
C14	0.079 (4)	0.053 (3)	0.079 (3)	−0.005 (3)	0.003 (3)	0.008 (3)
C15	0.046 (2)	0.035 (2)	0.050 (2)	0.0075 (18)	0.0084 (19)	0.0084 (18)
C16	0.045 (2)	0.039 (2)	0.058 (3)	0.0087 (19)	0.004 (2)	0.0093 (19)
C17	0.052 (3)	0.047 (3)	0.083 (3)	0.003 (2)	0.017 (2)	0.017 (2)
C18	0.068 (3)	0.052 (3)	0.077 (3)	0.006 (2)	0.015 (3)	0.032 (2)
C19	0.069 (3)	0.050 (3)	0.058 (3)	0.011 (2)	0.002 (2)	0.017 (2)
C20	0.083 (4)	0.095 (4)	0.098 (4)	0.016 (3)	−0.008 (3)	0.054 (3)
C21	0.047 (3)	0.090 (4)	0.128 (5)	0.012 (3)	0.003 (3)	0.041 (4)
I1	0.0894 (3)	0.0652 (2)	0.04841 (18)	0.00163 (17)	0.00875 (16)	0.01222 (15)
I2	0.0630 (2)	0.03952 (17)	0.0968 (3)	0.01431 (14)	0.00303 (17)	0.01007 (16)
I3	0.05512 (19)	0.0623 (2)	0.0677 (2)	0.01301 (14)	0.01072 (14)	0.03120 (15)
N1	0.064 (2)	0.057 (2)	0.050 (2)	0.0183 (19)	0.0019 (18)	0.0131 (18)
N2	0.057 (2)	0.056 (2)	0.057 (2)	0.0195 (18)	0.0023 (18)	0.0244 (18)
N3	0.049 (2)	0.045 (2)	0.061 (2)	0.0090 (16)	0.0021 (17)	0.0147 (17)
O1	0.079 (2)	0.066 (2)	0.076 (2)	0.0091 (18)	0.0111 (18)	0.0331 (18)
O2	0.0599 (19)	0.0423 (16)	0.0701 (19)	0.0027 (14)	0.0029 (16)	0.0033 (14)
O3	0.0402 (16)	0.069 (2)	0.087 (2)	0.0107 (15)	0.0047 (15)	0.0270 (17)

### Geometric parameters (Å, °)

**Table:**

C1—N1	1.323 (5)	C12—N2	1.348 (5)
C1—C2	1.392 (6)	C12—C13	1.496 (6)
C1—I1	2.110 (4)	C13—H13A	0.9600
C2—O1	1.355 (5)	C13—H13B	0.9600
C2—C3	1.390 (6)	C13—H13C	0.9600
C3—C4	1.366 (7)	C14—O2	1.426 (5)
C3—H3	0.9300	C14—H14A	0.9600
C4—C5	1.368 (7)	C14—H14B	0.9600
C4—H4	0.9300	C14—H14C	0.9600
C5—N1	1.324 (5)	C15—N3	1.324 (5)
C5—C6	1.497 (7)	C15—C16	1.382 (5)
C6—H6A	0.9600	C15—I3	2.114 (4)
C6—H6B	0.9600	C16—O3	1.359 (5)
C6—H6C	0.9600	C16—C17	1.380 (6)
C7—O1	1.449 (5)	C17—C18	1.369 (6)
C7—H7A	0.9600	C17—H17	0.9300
C7—H7B	0.9600	C18—C19	1.369 (6)
C7—H7C	0.9600	C18—H18	0.9300
C8—N2	1.315 (5)	C19—N3	1.357 (5)
C8—C9	1.389 (5)	C19—C20	1.506 (6)
C8—I2	2.115 (4)	C20—H20A	0.9600
C9—O2	1.356 (5)	C20—H20B	0.9600
C9—C10	1.394 (6)	C20—H20C	0.9600
C10—C11	1.367 (6)	C21—O3	1.438 (5)
C10—H10	0.9300	C21—H21A	0.9600
C11—C12	1.379 (6)	C21—H21B	0.9600
C11—H11	0.9300	C21—H21C	0.9600
			
N1—C1—C2	125.0 (4)	H13A—C13—H13B	109.5
N1—C1—I1	116.1 (3)	C12—C13—H13C	109.5
C2—C1—I1	118.9 (3)	H13A—C13—H13C	109.5
O1—C2—C3	126.1 (4)	H13B—C13—H13C	109.5
O1—C2—C1	118.2 (4)	O2—C14—H14A	109.5
C3—C2—C1	115.6 (5)	O2—C14—H14B	109.5
C4—C3—C2	118.8 (5)	H14A—C14—H14B	109.5
C4—C3—H3	120.6	O2—C14—H14C	109.5
C2—C3—H3	120.6	H14A—C14—H14C	109.5
C3—C4—C5	121.4 (5)	H14B—C14—H14C	109.5
C3—C4—H4	119.3	N3—C15—C16	124.5 (4)
C5—C4—H4	119.3	N3—C15—I3	115.2 (3)
N1—C5—C4	120.8 (5)	C16—C15—I3	120.3 (3)
N1—C5—C6	117.3 (5)	O3—C16—C17	126.2 (4)
C4—C5—C6	122.0 (5)	O3—C16—C15	117.2 (4)
C5—C6—H6A	109.5	C17—C16—C15	116.6 (4)
C5—C6—H6B	109.5	C18—C17—C16	119.8 (4)
H6A—C6—H6B	109.5	C18—C17—H17	120.1
C5—C6—H6C	109.5	C16—C17—H17	120.1
H6A—C6—H6C	109.5	C19—C18—C17	120.2 (4)
H6B—C6—H6C	109.5	C19—C18—H18	119.9
O1—C7—H7A	109.5	C17—C18—H18	119.9
O1—C7—H7B	109.5	N3—C19—C18	120.8 (4)
H7A—C7—H7B	109.5	N3—C19—C20	116.4 (4)
O1—C7—H7C	109.5	C18—C19—C20	122.8 (4)
H7A—C7—H7C	109.5	C19—C20—H20A	109.5
H7B—C7—H7C	109.5	C19—C20—H20B	109.5
N2—C8—C9	125.6 (4)	H20A—C20—H20B	109.5
N2—C8—I2	115.6 (3)	C19—C20—H20C	109.5
C9—C8—I2	118.8 (3)	H20A—C20—H20C	109.5
O2—C9—C8	118.2 (4)	H20B—C20—H20C	109.5
O2—C9—C10	125.8 (4)	O3—C21—H21A	109.5
C8—C9—C10	116.0 (4)	O3—C21—H21B	109.5
C11—C10—C9	118.9 (4)	H21A—C21—H21B	109.5
C11—C10—H10	120.6	O3—C21—H21C	109.5
C9—C10—H10	120.6	H21A—C21—H21C	109.5
C10—C11—C12	121.0 (4)	H21B—C21—H21C	109.5
C10—C11—H11	119.5	C1—N1—C5	118.3 (4)
C12—C11—H11	119.5	C8—N2—C12	117.8 (4)
N2—C12—C11	120.6 (4)	C15—N3—C19	118.0 (4)
N2—C12—C13	116.3 (4)	C2—O1—C7	116.9 (4)
C11—C12—C13	123.1 (4)	C9—O2—C14	117.1 (3)
C12—C13—H13A	109.5	C16—O3—C21	116.5 (4)
C12—C13—H13B	109.5		
			
N1—C1—C2—O1	179.0 (4)	C15—C16—C17—C18	−0.4 (6)
I1—C1—C2—O1	−2.2 (5)	C16—C17—C18—C19	−1.1 (7)
N1—C1—C2—C3	−0.6 (7)	C17—C18—C19—N3	1.5 (7)
I1—C1—C2—C3	178.2 (3)	C17—C18—C19—C20	−178.5 (5)
O1—C2—C3—C4	179.9 (5)	C2—C1—N1—C5	0.8 (6)
C1—C2—C3—C4	−0.5 (7)	I1—C1—N1—C5	−178.0 (3)
C2—C3—C4—C5	1.5 (8)	C4—C5—N1—C1	0.1 (7)
C3—C4—C5—N1	−1.3 (8)	C6—C5—N1—C1	−179.5 (4)
C3—C4—C5—C6	178.4 (5)	C9—C8—N2—C12	−1.1 (6)
N2—C8—C9—O2	−177.6 (4)	I2—C8—N2—C12	178.5 (3)
I2—C8—C9—O2	2.8 (5)	C11—C12—N2—C8	−1.1 (6)
N2—C8—C9—C10	2.5 (6)	C13—C12—N2—C8	178.6 (4)
I2—C8—C9—C10	−177.2 (3)	C16—C15—N3—C19	−1.5 (6)
O2—C9—C10—C11	178.5 (4)	I3—C15—N3—C19	−180.0 (3)
C8—C9—C10—C11	−1.6 (6)	C18—C19—N3—C15	−0.3 (6)
C9—C10—C11—C12	−0.4 (7)	C20—C19—N3—C15	179.8 (4)
C10—C11—C12—N2	1.8 (7)	C3—C2—O1—C7	−1.8 (7)
C10—C11—C12—C13	−177.9 (4)	C1—C2—O1—C7	178.7 (4)
N3—C15—C16—O3	−177.8 (3)	C8—C9—O2—C14	179.8 (4)
I3—C15—C16—O3	0.7 (5)	C10—C9—O2—C14	−0.3 (6)
N3—C15—C16—C17	1.8 (6)	C17—C16—O3—C21	−2.6 (6)
I3—C15—C16—C17	−179.7 (3)	C15—C16—O3—C21	177.0 (4)
O3—C16—C17—C18	179.1 (4)		

### Hydrogen-bond geometry (Å, °)

**Table:**

*D*—H···*A*	*D*—H	H···*A*	*D*···*A*	*D*—H···*A*
C14—H14B···O2^i^	0.96	2.56	3.429 (6)	151

Symmetry codes: (i) −*x*+2, −*y*+1, −*z*.
